# Traumatic posterior fossa extradural hematoma in children: a meta-analysis and institutional experience of its clinical course, treatment and outcomes

**DOI:** 10.1007/s10143-024-03089-2

**Published:** 2024-11-30

**Authors:** Keng Siang Lee, Shi Hui Ong, Conor S Gillespie, Lee Ping Ng, Wan Tew Seow, Sharon YY Low

**Affiliations:** 1 Department of Neurosurgery, King’s College Hospital, London, UK; 2 Department of Basic and Clinical Neurosciences, Maurice Wohl Clinical Neuroscience Institute, Institute of Psychiatry, Psychology and Neuroscience (IoPPN), King’s College London, London, UK; 3 Neurosurgical Service, KK Women’s and Children’s Hospital, Singapore, Singapore; 4 Department of Neurosurgery, Department of Clinical Neurosciences, University of Cambridge, Cambridge, UK; 5 Department of Neurosurgery, National Neuroscience Institute, Singapore, Singapore; 6 SingHealth Duke-NUS Neuroscience Academic Clinical Program, Singapore, Singapore; 7 SingHealth Duke-NUS Paediatrics Academic Clinical Program, Singapore, Singapore

**Keywords:** Extradural, Hematoma, Pediatric, Posterior fossa, Traumatic

## Abstract

Posterior fossa extradural hematoma (PFEDH) is rare but has a greater incidence amongst children. It is also associated with a rapid deterioration. The aim of this study was to present the management of PFEDH through our institutional experience and a meta-analysis. A retrospective single institution review of all children from 2004 to 2024 who underwent craniotomy for PFEDH was undertaken. The collected variables included: demographics, type of trauma, clinical findings, computed tomography findings, and clinical course. A systematic review using Ovid Medline, Ovid Embase, and Cochrane Central Register of Controlled Trials (CENTRAL), and meta-analysis were performed. Nineteen children with PFEDH who underwent surgery were identified. All 19 (100%) patients benefited from good Glasgow Outcome Scale (GOS) score 4–5, and there were no incidences of in-hospital mortality. From the systematic review, 391 patients, across twenty-four studies and our series, were included. A total of 308 were treated with surgery, whereas 83 patients were treated conservatively. A comparative meta-analysis was not performed as the two groups were deemed too heterogeneous in clinical characteristics. Instead, single-arm meta-analyses were performed. The pooled incidence of patients initially under conservative management requiring surgery was 9.90% (95%CI 1.61;22.21%, I^2^ = 35.2). The incidence of good functional outcomes in patients managed surgically and conservatively were 93.68% (95%CI: 88.69;97.57%, I^2^ = 0.0%), and 99.99% (95%CI: 96.53;100%, I^2^ = 0.0%), respectively. Overall pooled of mortality in patients managed surgically and conservatively were 0.57% (95%CI: 0.00;2.87%, I^2^ = 0.0%) and 0.00% (95%CI: 0.00;1.18%, I^2^ = 0.0%). Overall, our study reiterates that pediatric PFEDH is uncommon, and patients often present atypically. Based on our institutional experience and extrapolating data from our meta-analysis of the wider literature, neurosurgical intervention is a reliable therapeutic option with good clinical outcomes.

**Supplementary Information:**

The online version contains supplementary material available at 10.1007/s10143-024-03089-2.

## Introduction

Traumatic brain injury (TBI) in the pediatric population is a global cause of mortality and chronic disabilities; the latter imposing significant healthcare burden costs [1, 2]. Within the spectrum of TBI, posterior fossa extradural hematomas (PFEDHs) are rare and account for up to 3% of all extradural hematomas (EDH) [3–7]. PFEDH are more common in children [8, 9], and of significance, untreated cases are associated with rapid deterioration and demise [6]. However, PFEDH tends to present atypically – leading to the risks of dire consequences of a late or missed diagnosis [10]. Nonetheless, the widespread availability of computed tomography (CT) brain scans has led to better diagnostic capability, earlier neurosurgical intervention and overall, improved prognosis [5, 8, 11–14]. To date, most cases of traumatic PFEDH in children have been limited to case reports and small series [3–5, 7, 8, 11, 12, 15–31]. The aims of this study were hence twofold: (1) to present on the management of PFEDH through our institutional experience and (2) to corroborate our findings with the first meta-analysis of the published literature.

## Methods

### Data collection

This is an ethics-approved, retrospective study conducted at the KK Women’s and Children’s Hospital (SingHealth CIRB Reference: 2020/2632) with data obtained from 2008 to 2024. Patients less than 18 years old, with a diagnosis of traumatic PFEDH confirmed on neuroimaging that required intervention by the Neurosurgical Service, KK Women’s and Children’s Hospital, were included. Patients aged above 18 years old, diagnosed with supratentorial EDH, non-traumatic posterior fossa bleeds, and those with incomplete medical information were excluded. In addition, patients with known bleeding disorders and/or on long-term anticoagulation medication were also excluded. Individual patient information was either obtained from electronic data or hardcopy notes using a standardized data collection form. Imaging details for each patient were obtained from the radiology archives and assessed for completeness. The following variables were collected: patient demographics, Glasgow Coma Scale (GCS) and symptoms on admission, details of injury, the Pediatric Emergency Care Applied Research Network (PECARN) rule [32], radiological features on CT brain scans, and perioperative details of the intervention of choice (Table 1).

**Table 1:** Summary characteristics, clinical and radiological features and outcomes of children with PFEDH at our institution

Variables of interest	Number of patients (%)
Demographics	
Age*, years	5 (IQR 4–7)
Male	10 (52.6%)
Mechanism of head injury	
Fall	100 (100.0%)
Road traffic accident	0 (0.0%)
Interval between trauma and admission	
≤ 24 h of injury	11 (57.9%)
> 24 to 48 h of injury	3 (15.8%)
> 48 to 72 h of injury	5 (26.3%)
Presenting Glasgow Coma Scale (GCS)	
GCS 13 to 15	18 (94.7%)
GCS 9 to 12	1 (5.3%)
GCS 3 to 8	0 (0.0%)
Symptoms reported upon admission	
Headache	14 (73.7%)
Nausea	18 (94.7%)
Vomiting	18 (94.7%),
Transient loss of consciousness	0 (0.0%)
Drowsiness	4 (21.1%)
Seizures	0 (0.0%)
Asymptomatic	1 (5.3%)
Hematoma dimensions on CT brain scan	
Hematoma thickness > 15 ml	5 (26.3%)
Hematoma thickness 5 to 15 ml	14 (73.7%)
Hematoma thickness < 5 ml	0 (0.0%)
Hematoma volume (ml)	8.5 (IQR 5.7–13.2)
Other accompanying intracranial pathology on CT brain scan	
Occipital bone fracture	18 (100.0%)
Intraparenchymal contusion (ICH)	6 (31.6%)
Subdural hematoma (SDH)	3 (15.8%)
Subarachnoid hemorrhage (SAH)	1 (5.3%)
Supratentorial extension of primary hematoma	2 (10.5%)
Intraventricular hemorrhage (IVH)	0 (0.0%)
Pneumocephalus	2 (10.5%)
Mass effect and associated sequalae on CT brain scan	
Compression of fourth ventricle	15 (78.9%)
Obstructive hydrocephalus	16 (84.2%)
Obliteration of perimesencephalic cisterns	14 (73.7%)
Outcomes	
Good functional outcome GOS 4-5	19 (100.0%)
Good functional outcome GOS-E Peds 7–8	19 (100.0%)
Modified Rankins Score 0 (at 90 days post-surgery)	19 (100.%)
Death	0 (0.0%)

*Effect size reported as a median and interquartile range

Abbreviations: *CT* = computed tomography, *GCS* = Glasgow Coma Scale, *IVH* = intraventricular hematoma, LOC = loss of consciousness, RTA = road traffic accident, SAH = subarachnoid hemorrhage, SDH = subdural hematoma

### Overview of neurosurgical workflow and approaches

**Fig. 1:**
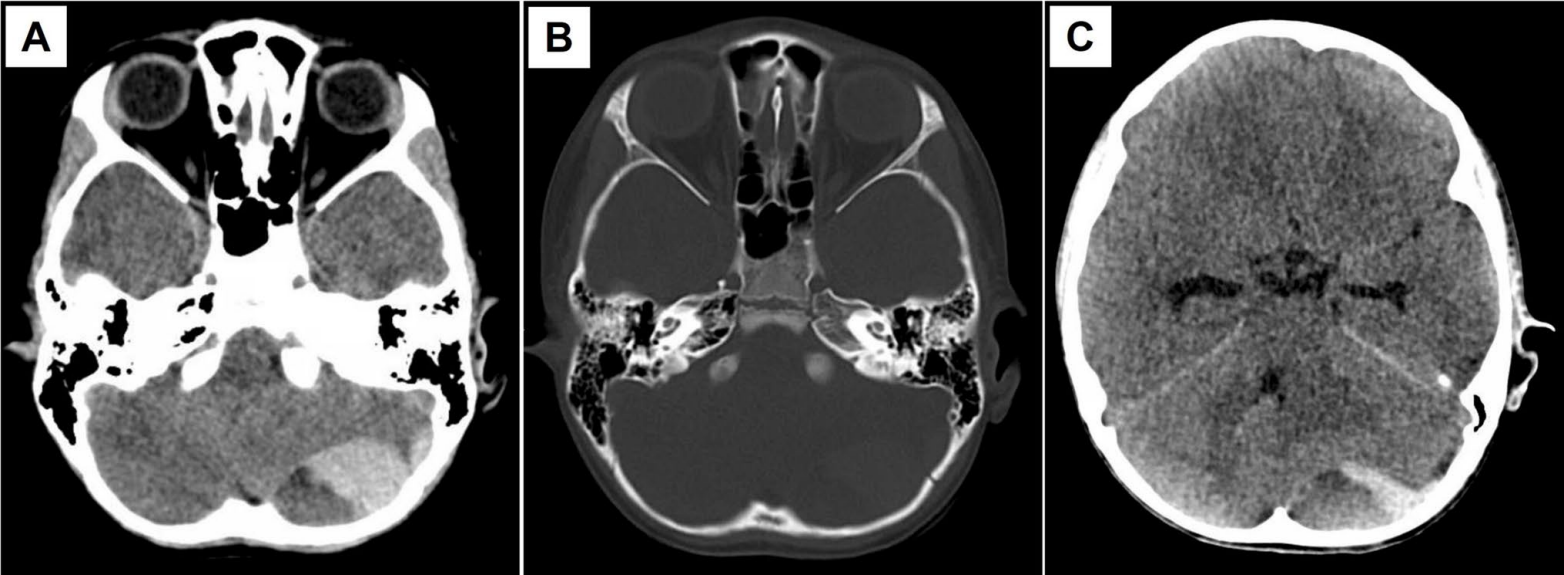
Case example of a pediatric patient with PFEDH from our series. Representative pre-operative CT brain images in axial direction depicting: (**A**) a left-sided, biconvex hematoma with mixed density causing local mass effect on the underlying parenchyma; (**B**) the corresponding bone window of (**A**) showing an occipital bone fracture adjacent to the PFEDH; (**C**) another image slice that shows radiological evidence of obstructive hydrocephalus, fourth ventricular distortion and effacement of the peri-mesencephalic cisterns. (Abbreviations: PFEDH = posterior fossa extradural hematoma; CT = computed tomography.)

The decision for intervention is in accordance with a standardized workflow for PFEDH. Briefly, a correlation between the clinical condition of the patients and CT scan images is confirmed. The main radiological feature of concern is an expansile clot—that is, PFEDH volume > 10ml, > 15 mm in thickness, with a midline shift of > 5 mm. Associated findings include obliteration of peri-mesencephalic cisterns, fourth ventricular displacement and obstructive hydrocephalus (Fig. 1).

The approach of choice is either a midline or paramedian suboccipital craniectomy or craniotomy [6]. For selected cases, the operating neurosurgeon may perform a single, large burrhole over the epicenter of the clot, especially if the CT scan demonstrates the clot to be more liquefied. Postoperatively, all patients are monitored overnight in either a high dependency or intensive care unit. We do not routinely perform postoperative CT scans if the patient has clinical improvement and adequate evacuation of the hematoma has been observed intraoperatively.

### Outcomes (case series)

The primary outcome measure was functional outcome assessed using the Glasgow Outcome Scale (GOS), GOS-Extended (GOS-E Peds) Score and 90-day modified Rankin Scale (mRS). The secondary outcome was in-hospital mortality [33–35].

### Statistical analysis

Numerical variables were described as median (interquartile range [IQR]) for non-normal distributions. Due to the modest sample size of our case series, descriptive analysis rather than statistical analyses to investigate associations, were performed to avoid the risk of a Type 2 false negative error.

Data were collated in Microsoft Excel (Microsoft, Redmond, WA, USA). All statistical analyses were performed using R software version 4.2.1 (R Foundation for Statistical Computing, 2022). *P*-values less than 0.05 were considered statistically significant.

### Systematic review and meta-analysis

This review was conducted according to the Preferred Reporting Items for Systematic Reviews and Meta-Analyses (PRISMA) guidelines [36]. The protocol was registered on the PROSPERO international prospective register of systematic reviews (registration number CRD42024557754) [37, 38].

### Outcomes (systematic review and meta analysis)

The primary outcome was good functional outcome defined as GOS 4–5. In studies where other outcome measures were used, such as the modified Rankin scale (mRS) or GOS-E Peds Score, they were translated into good or poor outcomes as defined above. For example, mRS 0–2 or GOS-E Peds 7–8 were translated into good functional outcomes. The secondary outcome was in-hospital mortality.

### Search strategy

Three electronic databases – Ovid Medline, Ovid Embase, and Cochrane Central Register of Controlled Trials (CENTRAL) – were searched. Searches were performed in each electronic database from its inception until 4th June 2024. In addition to its synonyms and related terms, concepts of “pediatric”, “posterior fossa” and “extradural hemorrhage” were used. Supplementary Table 1 presents the full search strategy used for each database.

### Eligibility criteria

Articles were selected for inclusion if they were either a primary interventional or observational study evaluating the management of PFEDH in an exclusively pediatric population (aged less than 18 years old). Additionally, we included mixed age population studies that provided analyses exclusive to the pediatric cohort. Supplementary Table 2 provides the full list of inclusion and exclusion criteria.

### Study selection

Titles and abstracts were independently screened against the pre-defined eligibility criteria developed by two reviewers (KSL and CSG). Any disagreements were resolved through discussion between the reviewers or further adjudication by a third reviewer (SYYL).

When encountered with multiple publications analyzing the same cohort over overlapping study periods, the publication that reported the largest patient data of relevant outcomes was used for evaluation.

### Data extraction

To ensure standardization and consistency, a proforma was developed and piloted to extract data on the following variables: (1) study details, (2) study design, (3) country and dataset, (4) selection criteria, (5) patient demographics, (6) treatment/control, (7) indication for treatment, and (8) results.

### Risk of bias assessment

The quality of included studies was assessed using the Joanna Briggs Institute (JBI) checklist for cohort studies and case series [39].

### Data synthesis

Meta-analyses were performed via the random effects model due to heterogeneity within and between individual studies as well as sampling variabilities across studies [39].

To obtain risk ratios (RRs) from reported binary outcomes, pairwise meta-analysis was conducted using the Mantel-Haenszel method without continuity correction, with the Paule-Mandel estimator. Overall pooled proportions of demographic comorbidities of included patients were computed with the generalized linear mixed model (GLMM) method using a random intercept logistic regression model via logit transformation [39]. Knapp-Hartung adjustments were used to reduce the chance of a false positive and to control the estimate uncertainties of between-study heterogeneity [39].

For pooling of means of numerical variables, we computed missing means and standard deviations (SDs) from medians, ranges (minimum to maximum) and interquartile ranges (IQRs) using the methods proposed by Hozo et al. and Wan et al. [40, 41].

The I^2^ statistic was employed to assess inter-study heterogeneity. I^2^ provides an estimate of the percentage of variability in results across studies that is due to real differences and not due to chance. I^2^ ≤ 30%, 30–50%, 50–75%, and ≥ 75% indicated low, moderate, substantial, and considerable heterogeneity, respectively. The quality of evidence for each outcome was evaluated using the GRADE framework [39, 42].

## Results

### Overview of study population and patient characteristics

A total of 157 pediatric head injury cases underwent neurosurgical intervention at our institution. Within this heterogeneous group, there were 19 (12.1%) cases of PFEDH (63.2% left side). There was no gender predilection (52.3% males) and the median age was 5 years (IQR 4–7 years). A fall from standing height was the most common mechanism of injury (57.9%).

Twelve (63.2%) patients presented to hospital only after 24 h of injury. Most patients had full GCS scores (84.2%) at the time of admission. The most common symptoms presented were nausea and vomiting (94.7%), headache (73.7%) and drowsiness (21.1%). No patients experienced a lucid interval.

The median volume of the hematoma was 8.5 ml (IQR 5.7–13.2 ml). Occipital bone fractures were observed in all 19 (100.0%) patients. Compression of the fourth ventricles, obliteration of the peri-mesencephalic cisterns and ventriculomegaly were observed in 15 (78.9%), 14 (73.7%), and 16 (84.2%) cases respectively.

Five (26.3%) and 14 (73.7%) patients underwent a burrhole or craniotomy respectively. At the time of surgery, all patients (100.0%) were seen to have bleeding from the occipital fracture lines. No mortality was observed in our series. All 19 (100.0%) patients had good recovery after a median hospital stay of 4 days (IQR 3.5–5 days), with good functional outcome at discharge. There were no surgical complications.

The length of follow-up ranged from 3 months to 8 years, and the median follow-up duration was 3 months (IQR 1.5–6.75 months). All patients reported good functional outcomes (GOS-E Peds8 and mRS0) on their last documented follow-up.

### Systematic review and meta-analysis

#### Characteristics of included studies

**Fig. 2:**
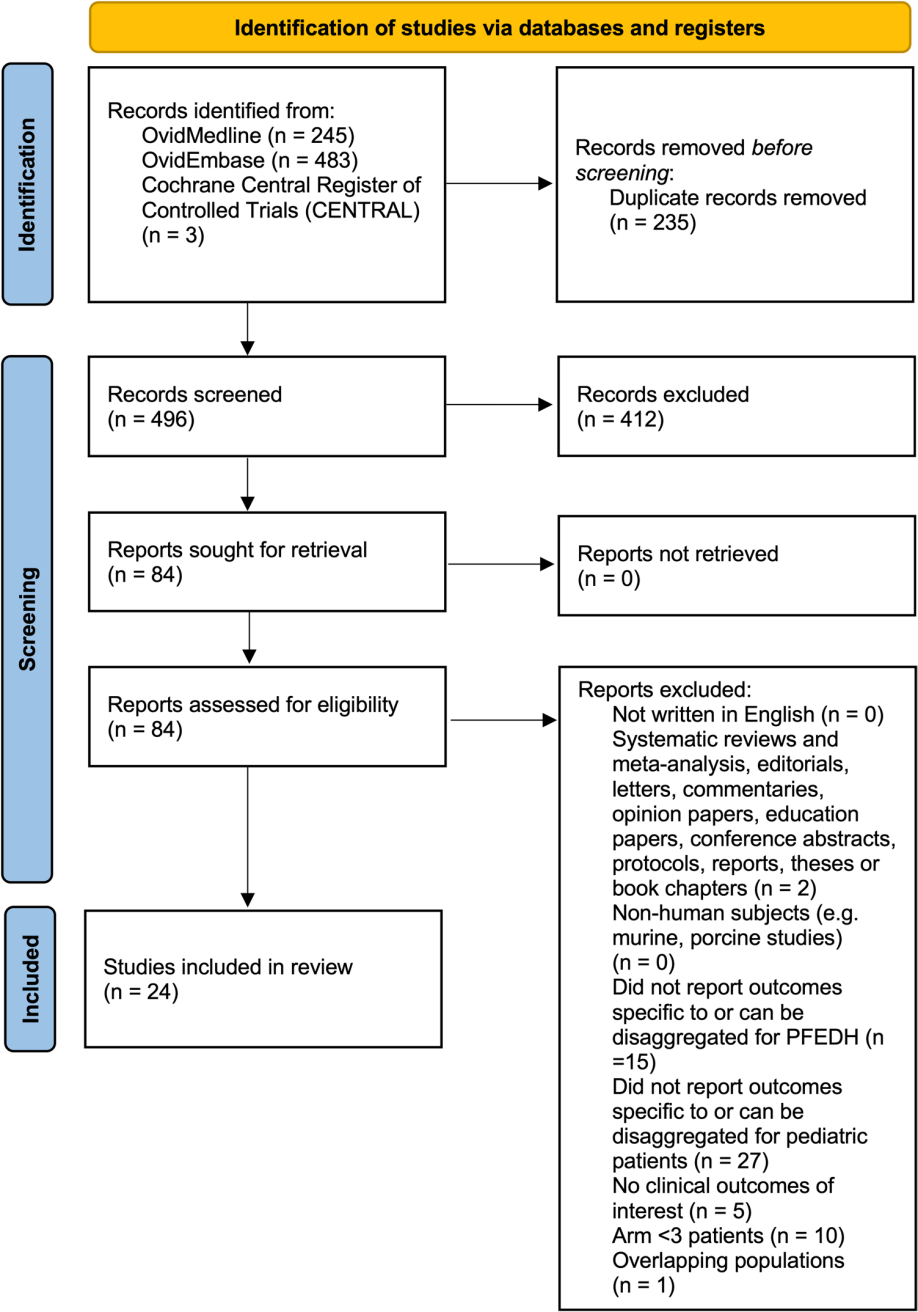
PRISMA flow diagram for studies included and excluded from the systematic review and meta-analysis

The systematic review search yielded 496 unique publications. Twenty-four studies including 372 pediatric patients met the eligibility criteria for inclusion in our meta-analysis (Fig. 2) [3–5, 7, 8, 11, 12, 15–31]. Including our institutional experience, a total of 391 pediatric patients were included.

All included studies were retrospective observational studies – five cohort studies and 19 case series. Table 2 presents a detailed summary of the characteristics and findings of the included studies. (Supplementary Tables 3 and 4 detail the risk of bias assessment.)

**Table 2:** Summary of included studies

First author and year	Country	Study design	Study period	Indications for surgery	Total patients included, *n*	Surgical, *n*	Conservative, *n*	Imputed mean age at surgery (SD), year*	Male, *n* (%)	Good function outcomes GOS4-5, *n* (%)	Death, *n* (%)
Ammirati M et al. 1984	USA	Case series	1976 to 1981	NR	4	4	NR	5.62 (3.49)	1 (25%)	4 (100%)	0 (0.0%)
Bellotti C et al. 1987	Italy	Case series	NR	Large hematomas and patient is symptomatic	5	4	1	10.00 (6.40)	4 (80%)	5 (100%)	0 (0.0%)
Berker M et al. 2003	Turkey	Case series	1971 to 2000	When the diagnosis is established, surgery should be performed as soon as possible even if the neurological examination is normal.	16	16	NR	7.74 (4.29)	8 (50%)	14 (87.5%)	1 (6.25%)
Bozbuga M et al. 1999	Turkey	Case series	1982 to 1992	Obliteration of the perimesencephalic cisterns, compression and/or displacement of the fourth ventricle, and the presence of hydrocephalus	9		9	NR	NR	9 (100%)	0 (0.0%)
Brambilla G et al. 1986	Italy	Case series	1979 to 1985	NR	4	4	NR	10.25 (5.31)	NR	3 (75%)	1 (25.0%)
Chaoguo Y et al. 2019	China	Cohort study	2001 to 2005	Clot volume of ≥15 ml or presence of fourth ventricle compression or displacement and/or obstructive ventriculomegaly	48	17	31	6.86 (4.55)	24 (50%)	46 (95.83%)	0 (0.0%)
Ciurea AV et al. 1993	Romania	Case series	1987 to 1992	Surgical treatment must be carried out immediately when the diagnosis has been established	9	9	NR	7.31 (2.89)	6 (66.67%)	8 (88.89%)	0 (0.0%)
Costa Clara JM et al. 1996	Spain	Case series	1989 to 1994	Once the diagnosis of a PFEDH is made, surgery is mandatory	3	3	NR	12.66 (5.77)	1 (33.33%)	3 (100%)	0 (0.0%)
Echara M et al. 2023	India	Case series	2016 to 2021	PFEDH ≥15 ml, or the presence of mass effect (compression of the fourth ventricle and/or hydrocephalus)	40	40	NR	15.23 (3.96)	32 (80%)	35 (87.5%)	1 (2.5%)
Ersahin Y et al. 1993	Turkey	Case series	1979 to 1991	Once the diagnosis of a PFEDH is made, the symptomatic hematomas are evacuated immediately	9	9	NR	8.46 (4.33)	5 (55.56%)	8 (88.89%)	1 (11.1%)
Garza-Mercado R et al. 1983	Mexico	Case series	NR	NR	6	6	NR	8.79 (6.14)	4 (66.67%)	5 (83.33%)	0 (0.0%)
Gupta PK et al. 2002	Oman	Case series	1992 to 2000	Hematoma > 10 ml in volume, > 15 mm in thickness, with a midline shift of > 5 mm, medullary compression and associated intracranial lesions	18	18	NR	NR	14 (77.78%)	15 (83.33%)	0 (0.0%)
Han K et al. 2018	China	Case series	2012 to 2015	Hematoma > 15 ml in volume	8	5	3	4.63 (1.06)	4 (50%)	8 (100%)	0 (0.0%)
Jamous MA et al. 2021	Jordan	Cohort study	2010 to 2021	Large hematoma thickness (≥ 15 mm) or the presence of fourth ventricle compression or obstructive ventriculomegaly or a GCS of < 14	16	7	9	7.70 (6.00)	12 (75%)	14 (87.5%)	1 (6.25%)
Jang JW et al. 2010	Korea	Cohort study	2001 to 2008	Large hematomas with mass effects, as well as associated intracranial lesions	19	9	10	7.94 (3.90)	10 (52.63%)	18 (94.74%)	0 (0.0%)
Koç RK et al. 1998	Turkey	Case series	1983 to 1994	NR	9	9	NR	9.44 (4.87)	5 (55.56%)	7 (77.78%)	2 (22.22%)
Lui TN et al. 1993	Taiwan	Case series	1977 to 1989	Once the diagnosis of a PFEDH is made, all are evacuated immediately	29	29	NR	NR	NR	NR	3 (10.34%)
Miao Z et al. 2023	China	Case series	2016 to 2021	Treatment with surgical evacuation depended on the clinical status of the patients as well as the presentation of CT image	40	40	NR	5.83 (1.08)	24 (60%)	32 (80%)	NR
Mori K et al. 1983	Japan	Case series	NR	Hematomas > 20 ml and patient is symptomatic	3	3	NR	5.66 (1.15)	1 (33.33%)	3 (100%)	0 (0.0%)
Pang D et al. 1983	USA	Case series	1978 to 1982	Clinical signs of local brain compression or herniation (such as increasing drowsiness, pupillary abnormality, hemiplegia, decerebrate posturing), or cardiorespiratory abnormalities	3	NR	3	7.33 (5.85)	NR	3 (100%)	0 (0.0%)
Peter JC et al. 1990	South Africa	Case series	1979 to 1989	NR	5	5	NR	7.00 (1.58)	2 (40%)	5 (100%)	NR
Prasad G et al. 2015	India	Cohort study	2008 to 2014	Hematoma with a volume of > 20 ml or radiological evidence of posterior fossa mass effect in the form of fourth ventricle distortion or compression and/or obstructive ventriculomegaly, irrespective of the GCS score	22	16	6	10.81 (4.64)	17 (77.27%)	20 (90.91%)	0 (0.0%)
Sencer A et al. 2012	Turkey	Cohort study	1995 to 2011	Hematoma thickness > 15 mm or hematoma thickness between 5–15 mm with injuries/ conditions (contusion, pneumocephalus, or SAH) causing additional mass effect or a GCS < 15	40	29	11	7.88 (4.67)	22 (55%)	40 (100%)	0 (0.0%)
Sheng HS et al. 2017	China	Case series	2010 to 2015	Criteria for standard craniectomy: hematoma volume > 30 ml or presence of severe mass effects like cerebellar tonsillar herniation Criteria for trephination mini-craniectomy: hematoma volume 10-30 ml, with mild mass effects (e.g. fourth ventricle compression or displacement and/or obstructive ventriculomegaly) and stable neurological conditions	7	7	NR	5.70 (2.32)	3 (42.86%)	7 (100%)	0 (0.0%)

Abbreviations: *NR* = Not reported, *UK* = United Kingdom, *USA* = United States

#### Patient baseline characteristics, treatment approach and radiological features

Pooled prevalence of baseline characteristics, stratified according to treatment arm, is summarized in Table 3. Of the 391 patients, 308 were treated with surgery, whereas 83 patients were treated conservatively.

**Table 3:** Summary of pooled baseline demographics, etiologies, clinical findings of included patients, CT brain findings and associated pathology between the two groups (surgical versus conservative)

Factor	Surgical cohort (*n* = 308)						Conservative cohort (*n* = 83)					
	Number of studies reporting this variable	Number of patients/ analysed	Pooled proportion [95% CI]	I^2^ (%)	*p*-value of I^2^ (from χ^2^ test)	Quality of Evidence (GRADE)	Number of studies reporting this variable	Number of patients/ analysed	Pooled proportion [95% CI]	I^2^ (%)	*p*-value of I^2^ (from χ^2^ test)	Quality of Evidence (GRADE)
Demographics												
Age*	16	183	7.78 [6.15; 9.41]	94.8	< 0.001	Low	3	16	6.61 [1.98; 11.24]	53.4	0.117	Low
Gender	17	204	61.51 [52.61; 70.11]	0.0	0.518	Low	3	19	54.13 [22.29; 84.56]	33.7	0.221	Low
Mechanism of injury												
Fall	17	215	75.44 [61.51; 87.48]	75.0	< 0.001	Low	4	49	92.70 [81.25; 99.61]	0.0	0.898	Low
RTA	14	182	31.73 [19.79; 44.74]	60.9	0.002	Low	4	49	7.30 [0.39; 18.75]	0.0	0.898	Low
Timing												
Within 24 h of injury	7	125	79.66 [69.68; 88.31]	24.3	0.244	Low	4	26	72.23 [50.65; 90.30]	11.5	0.335	Low
24–48 h of injury	4	60	28.69 [0.00; 78.98]	85.1	0.001	Low	2	20	19.48 [3.83; 41.05]	0.0	0.420	Low
48–72 h of injury	3	55	12.01 [0.00; 43.09]	84.0	0.002	Low	2	20	9.94 [0.01; 28.81]	0.0	0.870	Low
Preoperative GCS												
GCS13-15	14	196	65.84 [50.76; 79.64]	69.9	< 0.001	Low	6	70	100.00 [98.38; 100.00]	0.0	0.987	Low
GCS3-8	8	132	17.97 [8.61; 29.31]	49.1	0.056	Low	4	56	0.00 [0.00; 2.67]	0.0	0.946	Low
Symptoms												
Headache	20	266	63.55 [46.96; 78.89]	84.5	< 0.001	Low	7	73	57.02 [34.27; 78.60]	58.6	0.025	Low
Vomiting	20	266	77.69 [67.76; 86.51]	54.7	0.002	Low	7	73	46.59 [29.04; 64.50]	33.2	0.174	Low
Transient LOC	12	185	36.85 [19.59; 55.73]	78.2	< 0.001	Low	5	60	12.70 [0.00; 42.51]	78.6	0.001	Low
Drowsiness	12	131	29.49 [16.72; 43.71]	51.9	0.018	Low	5	58	7.24 [0.00; 35.66]	77.8	0.001	Low
Seizures	7	111	7.95 [0.89; 18.91]	50.3	0.060	Low	2	9	8.25 [0.00; 40.90]	0.0	0.519	Low
Asymptomatic	6	128	5.26 [0.00; 16.38]	73.9	0.002	Low	4	57	22.04 [11.19; 34.79]	0.0	0.979	Low
Signs												
Cerebellar signs	7	74	39.88 [19.87; 61.42]	55.3	0.037	Low	2	14	19.42 [0.83; 47.88]	0.0	0.550	Low
Babinski’s sign	4	22	40.64 [18.57; 64.37]	0.0	0.909	Low	NA	NA	NA	NA	NA	NA
Papilloedema	4	25	24.81 [2.42; 55.81]	46.1	0.135	Low	NA	NA	NA	NA	NA	NA
Aniscoria	3	74	7.75 [2.14; 15.65]	0.0	0.861	Low	NA	NA	NA	NA	NA	NA
Abducens palsy	5	31	24.27 [7.19; 45.54]	10.5	0.346	Low	NA	NA	NA	NA	NA	NA
Occipital swelling	5	60	44.22 [23.39; 65.97]	46.9	0.110	Low	NA	NA	NA	NA	NA	NA
Associated pathology												
Occipital bone fracture	20	257	76.30 [64.20; 86.89]	72.5	< 0.001	Low	5	67	66.88 [52.61; 79.93]	0.0	0.474	Low
Contusion	10	201	17.64 [10.62; 25.72]	34.6	0.131	Low	5	67	3.80 [0.01; 11.39]	0.0	0.723	Low
SDH	8	176	6.85 [2.72; 12.20]	5.0	0.391	Low	3	26	8.80 [0.00; 31.51]	41.8	0.180	Low
SAH	5	114	9.01 [3.85; 15.61]	0.0	0.905	Low	3	52	4.49 [0.01; 13.26]	0.0	0.626	Low
IVH	3	66	2.51 [0.00; 9.18]	19.4	0.289	Low	NA	NA	NA	NA	NA	NA
Pneumocephalus	6	118	9.41 [3.86; 16.42]	1.3	0.408	Low	2	21	3.18 [0.00; 17.75]	0.0	0.340	Low
CT findings												
Hematoma thickness > 15 ml	6	85	52.84 [32.12; 73.14]	65.1	0.014	Low	4	27	3.47 [0.00; 32.19]	57.8	0.069	Low
Hematoma thickness 5-15 ml	6	85	12.95 [0.00; 51.01]	88.3	< 0.001	Low	4	27	19.33 [0.00; 65.78]	81.5	0.001	Low
Hematoma thickness < 5 ml	6	85	56.94 [36.54; 76.33]	64.5	0.015	Low	3	24	49.83 [0.00; 100.00]	91.0	< 0.001	Low
Mean hematoma volume (ml)*	10	169	23.74 [16.19; 31.30]	93.5	< 0.001	Low	6	62	10.46 [6.16; 14.76]	87.9	< 0.001	Low
Mass effect												
Compression of fourth ventricle	11	122	49.95 [34.77; 65.13]	52.1	0.022	Low	3	54	0.00 [0.00; 1.60]	0.0	0.900	Low
Ventriculomegaly	10	116	36.10 [15.57; 59.06]	79.7	< 0.001	Low	4	54	0.00 [0.00; 2.93]	25.7	0.257	Low
Obliteration of persimencephalic cisterns	9	110	43.42 [21.53; 66.48]	77.0	< 0.001	Low	4	54	0.00 [0.00; 2.93]	25.7	0.257	Low

*Effect size is reported as a mean. Abbreviations: *CT* = computed tomography, *GCS* = Glasgow Coma Scale, *IVH* = intraventricular hematoma; LOC = loss of consciousness, *RTA* = road traffic accident, *SAH* = subarachnoid hemorrhage, *SDH* = subdural hematoma

The pooled proportion of males were 61.51% (95%CI: 52.61; 70.11%, I^2^ = 0.0% [*p* = 0.518]) % and 54.13% (95%CI: 22.29; 84.56%, I^2^ = 33.7% [*p* = 0.221]) in the surgical and conservative groups, respectively. Overall pooled mean age across the surgical and conservative groups were 7.78 years (95%CI: 6.15;9.41 years, I^2^ = 94.8%, *p* < 0.001) and 6.61 years (95%CI: 1.98;11.24 years, I^2^ = 53.4%, *p* = 0.117), respectively.

The most common symptoms encountered in the surgical group were headache (63.55% (95%CI: 46.96;78.89%, I^2^ = 84.5% [*p* < 0.001])) and vomiting (77.69% (95%CI: 67.76;86.51%, I^2^ = 54.7% [*p* = 0.002])). The most common symptoms encountered in the conservatively managed group were headache (57.02% (95%CI: 34.27;78.60%, I^2^ = 58.6% [*p* = 0.025])) and vomiting (46.59% (95%CI: 29.04;64.50%, I^2^ = 33.2% [*p* = 0.174])). Symptoms of transient loss of consciousness (LOC) (36.85% (95%CI: 19.59; 55.73%, I^2^ = 78.2% [*p* < 0.001]) versus 12.70% (95%CI: 0.00; 42.51%, I^2^ = 78.6% [*p* = 0.001])), drowsiness (29.49% (95%CI: 16.72; 43.71%, I^2^ = 51.9% [*p* = 0.018]) versus 7.24% (95%CI: 0.00;35.66%, I^2^ = 77.8% [*p* = 0.001])), and seizures (7.95% (95%CI: 0.89;18.91%, I^2^ = 50.3% [*p* = 0.060]) versus 8.25% (95%CI: 0.00;40.90%, I^2^ = 0.0% [*p* = 0.519])) were more common in the surgical group than the conservative group.

The pooled proportion of mass effective was greater in the surgical group than the conservative group – compression of the fourth ventricle (49.95% (95%CI: 34.77;65.13%, I^2^ = 52.1% [*p* = 0.022]) versus 8.25% (95%CI: 0.00;40.90%, I^2^ = 0.0% [*p* = 0.519])), ventriculomegaly (36.10% (95%CI: 15.57;59.06%, I^2^ = 79.7% [*p* < 0.001]) versus 8.25% (95%CI: 0.00;40.90%, I^2^ = 0.0% [*p* = 0.519])), obliteration of the perimesencephalic cisterns (43.42% (95%CI: 21.53;66.48%, I^2^ = 77.0% [*p* < 0.001]) versus 8.25% (95%CI: 0.00;40.90%, I^2^ = 0.0% [*p* = 0.519])). Following that, the pooled proportion of associated pathology was greater in the surgical group – occipital bone fracture (76.30% (95%CI: 64.20;86.89%, I^2^ = 72.5% [*p* < 0.001]) versus 66.88% (95%CI: 52.61;79.93%, I^2^ = 0.0% [*p* = 0.474])), contusion (17.64% (95%CI: 10.62;25.72%, I^2^ = 34.6% [*p* = 0.131]) versus 3.80% (95%CI: 0.01;11.39%, I^2^ = 0.0% [*p* = 0.723])) and pneumocephalus (9.41% (95%CI: 3.86;16.42%, I^2^ = 1.3% [*p* = 0.408]) versus 3.18% (95%CI: 0.00;17.75%, I^2^ = 0.0% [*p* = 0.340])).

The mean hematoma volume was greater in the surgical group (23.74 ml (95%CI: 16.19;31.30 ml, I^2^ = 93.5%, *p* < 0.001)) compared with the conservatively managed group (10.46 ml (95%CI: 6.16;14.76 ml, I^2^ = 87.9%, *p* < 0.001)) (Table 3).

#### Conversion to surgery when initially managed conservatively

Conversion to surgery from conservative management was reported in five studies across 77 patients initially managed conservatively. Reasons cited were enlargement of the hematoma and/or clinical deterioration. The overall pooled incidence of conversion was 9.90% (95%CI: 1.61;22.21%, I^2^ = 35.2%, *p* = 0.187).

#### Rates of good functional outcomes and mortality

**Fig. 3:**
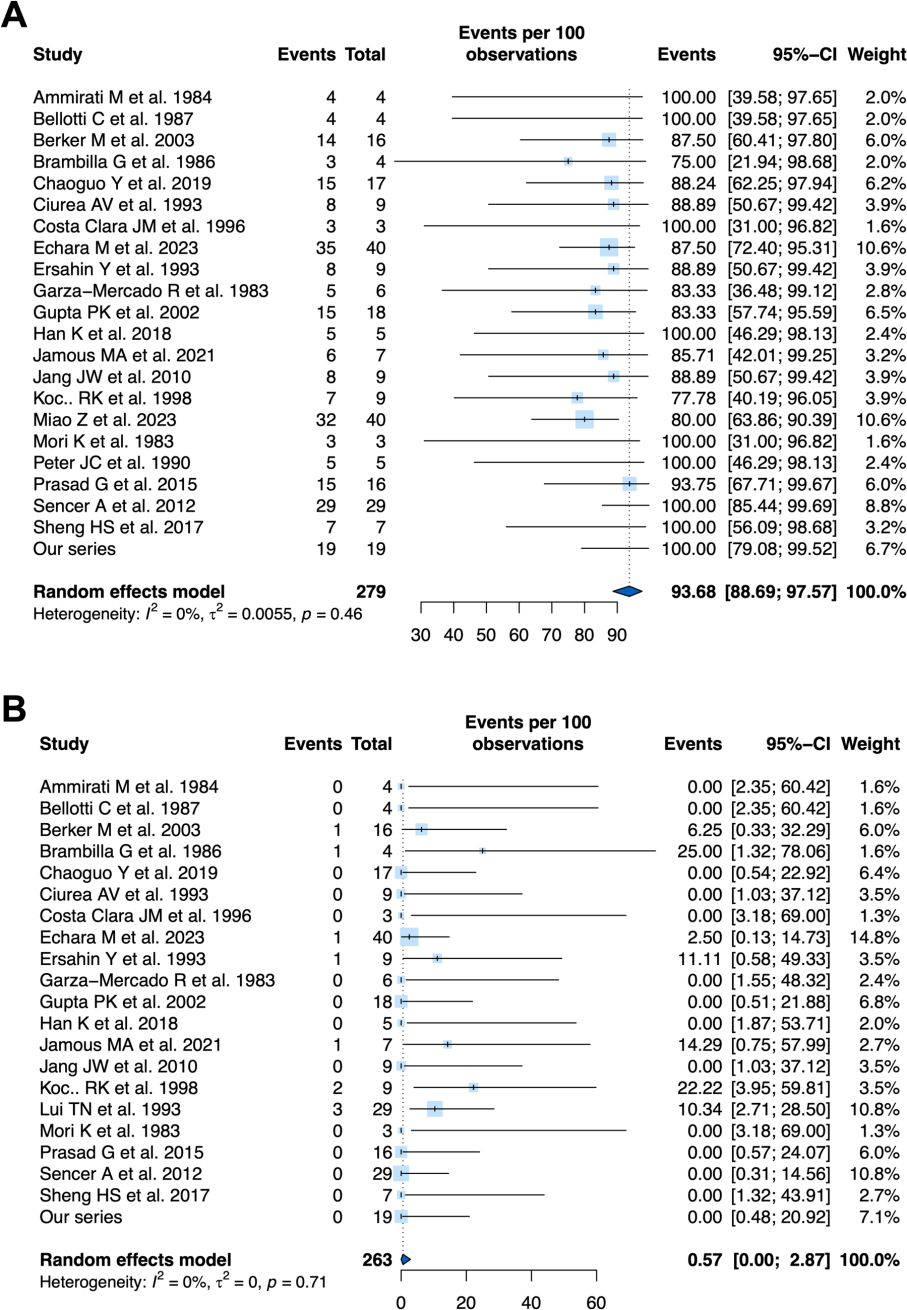
Forest plots, with random-effects model, of pooled percentage of (**A**) good functional outcome (GOS 4–5), and (**B**), in-hospital mortality, in patients PFEDH who underwent surgery, respectively

A comparative meta-analysis of outcomes was not performed as the two groups were deemed too heterogeneous in terms of baseline clinical characteristics. Instead, single-arm meta-analyses were performed (Table 4). The overall pooled proportion of good functional outcomes across 279 patients between these studies, and our series, managed surgically was 93.68% (95%CI: 88.69;97.57%, I^2^ = 0.0% [*p* = 0.46]) (Fig. 3). The incidence of good functional outcomes was reported in eight studies assessing 82 patients managed conservatively. Overall pooled incidence was 99.99% (95%CI: 96.53,100%, I^2^ = 0.0% [*p* = 0.62]) (Supplementary Fig. 1). The overall pooled incidence of mortality across 263 patients between these studies, and our series, managed surgically was 0.57% (95%CI: 0.00;2.87%, I^2^ = 0.0% [*p* = 0.71]). The incidence of mortality was reported in eight studies assessing 82 patients managed conservatively. Overall pooled incidence was 0.00% (95%CI: 0.00,1.18%, I^2^ = 0.0% [*p* = 0.99]).

**Table 4:** Pooled outcomes of included patients between the two groups (surgical versus conservative)

Outcomes	Surgical (*n* = 308)						Conservative (*n* = 83)					
	No. of studies reporting variable	No. of patients/ analyzed	Pooled proportion [95% confidence interval]	I^2^ (%)	*P* value of I^2^ (from χ^2^ test)	Quality of Evidence (GRADE)	No. of studies reporting variable	No. of patients/ analyzed	Pooled proportion [95% confidence interval]	I^2^ (%)	*P* value of I^2^ (from χ^2^ test)	Quality of Evidence (GRADE)
Good functional outcome	22	279	93.68 [88.69; 97.57]	0.0	0.461	Low	8	82	99.99 [96.53; 100.00]	0.0	0.624	Low
Death	21	263	0.57 [0.00; 2.87]	0.0	0.706	Low	8	82	0.00 [0.00; 1.18]	0.0	0.997	Low

## Discussion

### Overview of PFEDH in the pediatric population

Amidst the spectrum of head injuries treated by neurosurgeons, the entity of PFEDHs is relatively uncommon [11, 14, 15, 17]. Nonetheless, a late diagnosis is associated with a high mortality rate [14]. Despite published studies reporting a higher incidence of PFEDH in children in comparison to adults, children tend to fare better with prompt intervention [8, 9, 30]. PFEDHs are usually associated with occipital bone fractures. Here, the anatomical disadvantage is that children tend to have larger occipital sinuses; hence, torrential venous bleeding may be encountered in fractures crossing the midline with tearing of adjacent venous sinuses. Under such circumstances, bleeding leads to progressive stripping of the dura from the inner table of the skull [43]. This expanding PFEDH could be rapidly fatal due to the compromise of a smaller posterior cranial fossa, leading to brainstem compression, tonsillar herniation and obstructive hydrocephalus [3, 8, 44].

Specifically for PFEDH, one of the main challenges is that affected patients are likely to present in an insidious fashion and may not manifest the classic textbook description of an initial loss of consciousness followed by a lucid interval, subsequently developing an ipsilateral pupillary dilation and progressive obtundation [10, 45]. Clinical assessment in young children is often difficult due to their variable, non-specific complaints, especially in the preverbal age group [46–48]. Owing to their smaller posterior fossa, precipitous drops in the neurological status may occur without forewarning [6, 43]. Congruently, we observed that most patients had full GCS and no focal neurological deficit in our cohort, at the time of admission.

Following that, headache, and or vomiting are the most common presenting complaints in our study, aligning with other publications [3, 5, 11, 15, 23]. Therefore, a diagnosis of PFEDH should be considered if there is a history of headache, vomiting and trauma to the occipital region, even for relatively stable GCS on admission. In corroboration with existing studies, we practiced a low threshold for neurosurgical intervention. This is to mitigate the risk of sudden and rapid clinical deterioration, especially for symptomatic patients with existing radiological evidence of mass effect [7, 17].

### Imaging characteristics in PFEDH

The current gold standard for neuroimaging in TBI is a CT brain to reliably exclude any life-threatening intracranial hemorrhage [11, 49]. The impact of an early CT upon the treatment of PFEDH is easily recognized—that is, diagnosis can be established quickly to initiate intervention; hence, affording better prognosis [5]. As mentioned previously, occipital linear fractures are common in up to 80% of cases of PFEDH. Occasionally, the hematoma may be undetected on the initial CT scan if the scan is performed too early because PFEDHs tend to appear less dense [13]. This is due to the fact that the bleed tends to be venous and liquefies earlier than supratentorial hematomas [50]. Extension of the PFEDH to the supratentorial compartment is another common radiological finding [4]. In our cohort, we noted two (10.5%) had such extensions, comparable to other reports [4, 23, 51]. The presence of obstructive hydrocephalus is a poor prognosticator [3, 8, 14, 15, 23, 26, 52–54]. Presence of other accompanying lesions such as contusions, subdural hematomas, subarachnoid hemorrhage, intraventricular bleeds and pneumocephalus have been reported [3–5, 7, 10, 11, 23]. In our study, 16 patients (84.2%) had radiological features of early obstructive hydrocephalus and six (31.6%) had other accompanying bleeds and/or pneumocephalus. Nonetheless, we did not observe a poorer outcome for the subgroup of patients.

### Towards an evidence-based management strategy

As reflected in our population, time lapse from injury may be delayed up to a few days from initial injury [17]. Early diagnosis relies on astute clinical observations and failure to recognize the more subtle clinical presentation in children is a genuine concern [10]. To mitigate this, some authors have suggested serial control scans at various time-points post-TBI, for cases where the clinical history is suspicious of PFEDH even if no bleed is obvious on earlier imaging [5, 11]. We are cognizant that most clinicians are mindful of exposing young patients to excessive ionizing radiation in view of the risks of future malignancies [55–58]. Other pertinent challenges associated with CT scans include the possible need for sedation during the scan and parental preferences [58]. Levelling on these opposing points, we advocate that an early CT scan is a reasonable option for selected patients versus a more conservative approach, considering the potential for rapid deterioration. Interestingly, we also observe the limitations of the PECARN rule in our series. This is a prediction score used to assess the need for CT scans children with a history of minor head trauma that has been validated in large, multi-centred studies – the PECARN rule provides the highest level of internationally valid guidance available [59, 60]. However, here, only four patients (21%) of our children underwent initial CT scan based on PECARN predictions. We hypothesize that the role of PECARN may have a limited negative predictive value in this specific cohort of patients with PFEDH, due to the initial subtle nature of PFEDH presentation. This limited negative predictive value may be attributed to the fact that the PECARN rule was derived from a wider population that included a spectrum of traumatic head injuries of varying severity [59]. With the limited utility of the PECARN in PFEDH, the infrequent occurrence of PFEDH and its extended history of evolution from comparatively low-impact fall mechanisms, affected children may initially be cleared with false reassurance – leading to false negative cases with dire consequences. A revised sub-criteria to recognize their diagnosis early may be more suitable for them. However, this requires input from international workgroups and external validation in future studies.

The traditional treatment of PFEDH is surgical evacuation. Balancing the of risk of a rapid deterioration with conservative management versus performing a relatively safe prophylactic surgery, surgery has conventionally been recommended even if the child is initially asymptomatic [4, 43]. However, no surgery is risk-free and torrential venous bleeding may be encountered intraoperatively especially with associated fractures crossing the midline with tearing of adjacent venous sinuses. To date, there is no clear, defined criteria for deciding between conservative and surgical treatment [5, 13, 17]. We observe the following CT brain characteristics are often used: hematoma > 10 ml in volume or > 15 mm in thickness, with a midline shift of > 5 mm, or mass effect of peri-mesencephalic cisterns obliteration, fourth ventricle displacement, and or presence of hydrocephalus [5, 14, 21, 23, 52, 53, 61]. Generally, most studies emphasize that enlarging hematomas and neurological deterioration are urgent indications for surgery due to the risk of rapid compromise in the posterior fossa. In our meta-analysis, a fifth of patients were managed conservatively with close neuro-monitoring and serial imaging, whilst the rest were treated surgically. The prognosis of PFEDH is reported to be good [3–5, 7, 8, 11, 12, 15–31]. Regardless of either approach, studies generally report good prognosis in pediatric PFEDH [3–5, 7, 10, 11, 15, 19, 21, 23]. In addition, the rate of good postoperative outcomes imply that surgery does not further morbidity or mortality to the existing neurological state. For the purposes of our study, we did not perform a comparative meta-analysis between the conservative and surgical cohorts as the clinical characteristics were too heterogeneous between both groups. In addition, an established criteria for either of the treatments remain to be elucidated, which we have summarized in Table 2 [3–5, 7, 8, 11, 12, 15–31]. Future research could address this current paucity in evidence with direct head-to-head comparisons.

### Limitations and future directions

Owing to its rare entity, the pediatric cases of traumatic PFEDH published in the literature are limited to case reports and small series. Henceforth, the limitations of our meta-analysis stem from inclusion of small retrospective observational studies and the heterogeneity across them [62–64]. Restricted by the modest number of suitable studies and small number of events, our ability to delineate risk factors for poor outcomes was also limited, largely due to the risk of Type 2 false negative errors. Nonetheless, congruent findings from our study and meta-analysis do demonstrate the effectiveness of neurosurgical intervention in children with PFEDH, as long as they are interpreted judiciously with the aforementioned limitations.

Separately, we want to highlight that patients in this age group tend to live longer, and significant brain trauma leads to long-term consequences that may permanently compromise their motor development and cognitive abilities [2]. The acquired injury in a developing brain can disrupt subsequent its development, with delayed consequences emerging over time, causing significant short- and long-term alterations in a wide range of functional abilities [65–67]. Longitudinal studies have reported that most children experience persistent disability at one year after severe TBI, especially in the cognitive, behavioural, emotional and social domains [67, 68]. Building on this knowledge, we recommend future studies to consider the long-term quality of life and the major factors limiting the resumption of daily activities in PFEDH patients [14].

## Conclusion

Overall, our study reiterates that pediatric PFEDH is uncommon, and patients often present atypically. Based on our institutional experience and extrapolating data from our meta-analysis of the wider literature, neurosurgical intervention is a reliable therapeutic option with good clinical outcomes. Emphasis is on early recognition of their subtle symptoms by mindful clinicians to initiate early neuroimaging. Future multi-centre collaborations may be warranted to propose a standardized criterion for conservative management of PFEDH. As the way forward, we advocate continued efforts to educate the public on pediatric head injury prevention.

## Supplementary Information

Below is the link to the electronic supplementary material.

ESM 1 (DOCX 43.1 KB)

## Data Availability

No datasets were generated or analysed during the current study.
