# Supplementary material for: Traumatic posterior fossa extradural hematoma in children: a meta-analysis and institutional experience of its clinical course, treatment and outcomes
Source: Neurosurg Rev. 2024 Nov 30;47(1):878. doi: 10.1007/s10143-024-03089-2 (PMC11608393; doi:10.1007/s10143-024-03089-2)
Supplement: Supplementary file 1 — (DOCX 43.1 KB) [file 10143_2024_3089_MOESM1_ESM.docx]

Supplementary Table 1. Search strategy used for the three electronic databases (up to 04 June 2024)

| **EMBASE search** | | **483 articles** |
| --- | --- | --- |
| No. | Search term | |
| **Posterior fossa concept** | | |
| 1 | exp posterior fossa/ | |
| 2 | (Posterior fossa or infratentorial).tw. | |
| 3 | 1 or 2 | |
| **Extradural hematoma concept** | | |
| 4 | exp epidural hematoma/ | |
| 5 | exp epidural space/ | |
| 6 | exp brain hematoma/ | |
| 7 | 5 and 6 | |
| 8 | (Extradural hematoma or epidural hematoma).tw. | |
| 9 | 4 or 7 or 8 | |
| **Combined concepts** | | |
| 10 | 3 and 9 | |

| **Medline search** | | **245 articles** |
| --- | --- | --- |
| No. | Search term | |
| **Posterior fossa concept** | | |
| 1 | (Posterior fossa or infratentorial).tw. | |
| **Extradural hematoma concept** | | |
| 2 | exp Hematoma, Epidural, Cranial/ | |
| 3 | exp Epidural Space/ | |
| 4 | exp Cerebral Hemorrhage/ or exp Hematoma/ | |
| 5 | 3 and 4 | |
| 6 | (Extradural hematoma or epidural hematoma).tw. | |
| 7 | 2 or 5 or 6 | |
| **Combined concepts** | | |
| 8 | 1 and 7 | |

| **Cochrane Central Register of Controlled Trials (CENTRAL**) | | **3 articles** |
| --- | --- | --- |
| No. | Search term | |
| **Posterior fossa concept** | | |
| 1 | (Posterior fossa or infratentorial):ti,ab,kw | |
| **Extradural hematoma concept** | | |
| 2 | MeSH descriptor: [Hematoma, Epidural, Cranial] explode all trees | |
| 3 | MeSH descriptor: [Epidural Space] explode all trees | |
| 4 | MeSH descriptor: [Cerebral Hemorrhage] explode all trees | |
| 5 | MeSH descriptor: [Hematoma] explode all trees | |
| 6 | #4 or #5 | |
| 7 | #3 and #6 | |
| 8 | (Extradural hematoma or epidural hematoma):ti,ab,kw | |
| 9 | #2 or #7 or #8 | |
| **Combined concepts** | | |
| 10 | #1 and #9 | |

Supplementary Table 2. Inclusion and exclusion criteria used to select studies for the review

| Inclusion criteria | Exclusion criteria |
| --- | --- |
| Primary interventional or observational studies assessing management of posterior fossa extradural hematoma in children (aged <19 years) | - Not written in English - Systematic reviews and meta-analysis, editorials, commentaries, opinion papers, letters, education papers, conference abstracts, protocols, reports, theses or book chapters - Treatment not tested in the clinical setting (e.g. lab based rather than clinical practice) - Non-human subjects (e.g. murine, porcine studies) - Outcomes not specific to posterior fossa extradural hematoma - Outcomes not specific to pediatric patients (aged <18 years) - Overlapping populations - Arm <3 patients - Did not report outcomes of interests, or meaningful extractable data |

Supplementary Table 3. Joanna Briggs Institute quality assessment checklist for case series.

|  |  | | **Question no.** | | | | | | | | |  |
| --- | --- | --- | --- | --- | --- | --- | --- | --- | --- | --- | --- | --- |
| **Study** | **1** | **2** | | **3** | **4** | **5** | **6** | **7** | **8** | **9** | **10** | **Overall** |
| Ammirati M et al. 1984 |  | ✔ | | ✔ |  |  | ✔ | ✔ | ✔ | ✔ | ✔ | 7 |
| Bellotti C et al. 1987 |  | ✔ | | ✔ | ✔ | ✔ | ✔ | ✔ | ✔ | ✔ | ✔ | 9 |
| Berker M et al. 2003 | ✔ | ✔ | | ✔ | ✔ | ✔ | ✔ | ✔ | ✔ | ✔ | ✔ | 10 |
| Bozbuga M et al. 1999 | ✔ | ✔ | | ✔ | ✔ | ✔ | ✔ | ✔ | ✔ | ✔ | ✔ | 10 |
| Brambilla G et al. 1986 | ✔ | ✔ | | ✔ | ✔ | ✔ | ✔ | ✔ | ✔ | ✔ | ✔ | 10 |
| Ciurea AV et al. 1993 | ✔ | ✔ | | ✔ | ✔ | ✔ | ✔ | ✔ | ✔ | ✔ | ✔ | 10 |
| Costa Clara JM et al. 1996 | ✔ | ✔ | | ✔ | ✔ | ✔ | ✔ | ✔ | ✔ | ✔ | ✔ | 10 |
| Echara M et al. 2023 | ✔ | ✔ | | ✔ | ✔ | ✔ | ✔ | ✔ | ✔ | ✔ | ✔ | 10 |
| Ersahin Y et al. 1993 | ✔ | ✔ | | ✔ | ✔ | ✔ | ✔ | ✔ | ✔ | ✔ | ✔ | 10 |
| Garza-Mercado R et al. 1983 | ✔ | ✔ | | ✔ | ✔ | ✔ | ✔ | ✔ | ✔ | ✔ | ✔ | 10 |
| Gupta PK et al. 2002 | ✔ | ✔ | | ✔ |  |  | ✔ | ✔ | ✔ | ✔ | ✔ | 8 |
| Han K et al. 2018 | ✔ | ✔ | | ✔ | ✔ | ✔ | ✔ | ✔ | ✔ | ✔ | ✔ | 10 |
| Koç RK et al. 1998 | ✔ | ✔ | | ✔ | ✔ | ✔ | ✔ | ✔ | ✔ | ✔ | ✔ | 10 |
| Lui TN et al. 1993 | ✔ | ✔ | | ✔ | ✔ | ✔ | ✔ | ✔ | ✔ | ✔ | ✔ | 10 |
| Miao Z et al. 2023 | ✔ | ✔ | | ✔ | ✔ | ✔ | ✔ | ✔ | ✔ | ✔ | ✔ | 10 |
| Mori K et al. 1983 | ✔ | ✔ | | ✔ | ✔ | ✔ | ✔ | ✔ | ✔ | ✔ | ✔ | 10 |
| Pang D et al. 1983 | ✔ | ✔ | | ✔ | ✔ | ✔ | ✔ | ✔ | ✔ | ✔ | ✔ | 10 |
| Peter JC et al. 1990 | ✔ | ✔ | | ✔ | ✔ | ✔ | ✔ | ✔ | ✔ | ✔ | ✔ | 10 |
| Sheng HS et al. 2017 | ✔ | ✔ | | ✔ | ✔ | ✔ | ✔ | ✔ | ✔ | ✔ | ✔ | 10 |

1. Were there clear criteria for inclusion in the case series?

2. Was the condition measured in a standard, reliable way for all participants included in the

case series?

3. Were valid methods used for identification of the condition for all participants included in the

case series?

4. Did the case series have consecutive inclusion of participants?

5. Did the case series have complete inclusion of participants?

6. Was there clear reporting of the demographics of the participants in the study?

7. Was there clear reporting of clinical information of the participants?

8. Were the outcomes or follow up results of cases clearly reported?

9. Was there clear reporting of the presenting site(s)/clinic(s) demographic information?

10. Was statistical analysis appropriate?

Supplementary Table S4. Joanna Briggs Institute quality assessment checklist for cohort studies

|  |  | | **Question no.** | | | | | | | | | |  | |  | |
| --- | --- | --- | --- | --- | --- | --- | --- | --- | --- | --- | --- | --- | --- | --- | --- | --- |
| **Study** | **1** | **2** | | **3** | **4** | **5** | **6** | **7** | **8** | **9** | **10** | **11** | | **Overall** | |  |
| Chaoguo Y et al. 2019 |  | ✔ | | ✔ |  |  | ✔ | ✔ | ✔ | ✔ | ✔ | ✔ | | 8 | |  |
| Jamous MA et al. 2021 |  | ✔ | | ✔ |  |  | ✔ | ✔ | ✔ | ✔ | ✔ | ✔ | | 8 | |  |
| Jang JW et al. 2010 |  | ✔ | | ✔ |  |  | ✔ | ✔ | ✔ | ✔ | ✔ | ✔ | | 8 | |  |
| Prasad G et al. 2015 |  | ✔ | | ✔ |  |  | ✔ | ✔ | ✔ | ✔ | ✔ | ✔ | | 8 | |  |
| Sencer A et al. 2012 |  | ✔ | | ✔ |  |  | ✔ | ✔ | ✔ | ✔ | ✔ | ✔ | | 8 | |  |

1. Were the two groups similar and recruited from the same population?

2. Were the exposures measured similarly to assign people to both exposed and unexposed groups?

3. Was the exposure measured in a valid and reliable way?

4. Were confounding factors identified?

5. Were strategies to deal with confounding factors stated?

6. Were the groups/participants free of the outcome at the start of the study (or at the moment of exposure)?

7. Were the outcomes measured in a valid and reliable way?

8. Was the follow up time reported and sufficient to be long enough for outcomes to occur?

9. Was follow up complete, and if not, were the reasons to loss to follow up described and explored

10. Were strategies to address incomplete follow up utilized?

11. Was appropriate statistical analysis used?

Supplementary Figure 1. Forest plot, with random-effects model, of the pooled percentage of good functional outcome (GOS 4-5), in patients PFEDH who were managed conservatively.
